# Identifying What’s Vital in Shift Handover: The ABC-VITAL Model for ED Transitions

**DOI:** 10.7759/cureus.96860

**Published:** 2025-11-14

**Authors:** Albert J Peñaloza, Jovanny Garcés, Claudia Salazar, Ana Cadavid

**Affiliations:** 1 Emergency Medicine, Universidad Cooperativa de Colombia, Medellín, COL; 2 Epidemiology, Fundación Universitaria del Área Andina, Bogotá, COL; 3 Emergency Medicine, Clínica Las Vegas, Medellín, COL

**Keywords:** emergency department, health communication, leadership, patient handoff, patient safety

## Abstract

Shift handover in EDs is a critical event that directly impacts patient safety, care continuity, and staff well-being. This technical report introduces the ABC-VITAL model of shift handover, structured around three essential components: Assistance, Balance, and Closure. The model was developed from the convergence of healthcare management, emergency medicine, and academic leadership and is designed to be adaptable across different levels of complexity and clinical profiles. The Assistance component is based on the ID-VITAL framework, which supports structured, decision-oriented clinical communication. The Balance component addresses the emotional and interpersonal aspects of handover, promoting team cohesion and a psychologically safe work environment. The Closure component promotes operational anticipation and team synchronization to guide the incoming team’s operational planning. This model reframes the shift handover as a clinical, human, and strategic process, with the potential to influence organizational culture in emergency care settings.

## Introduction

Effective shift handover in EDs is a cornerstone of patient safety and operational continuity. Inadequate communication during transitions of care has been associated with adverse events, treatment delays, and increased cognitive load among clinicians [1,2]. These challenges are intensified in high-pressure ED environments, where time-sensitive decisions, multitasking, and team fragmentation are common [3].

Handover is not merely a biomedical information exchange; it encompasses leadership, team synchronization, pending interventions, and emotional closure [2,4,5]. Existing frameworks have demonstrated improvements in clinical efficiency and communication, yet many tend to overlook broader operational and human dimensions involved in transitions of care [3,5,6].

This report presents the ABC-VITAL model for ED shift handover, which integrates three key dimensions: Assistance (A), Balance (B), and Closure (C). Each component addresses critical aspects of shift transitions. “Assistance” focuses on clinical data and includes the operational format ID-VITAL; “Balance” incorporates elements of staff readiness, emotional stability, and shift-level situational awareness; and “Closure” emphasizes mutual understanding, agreed disposition plans, and team synchronization for the incoming shift [2,3,6-8].

The ID-VITAL mnemonic within the Assistance component provides a structured, reproducible checklist for handoff communication. It includes Identification, Diagnosis, Vital Signs, Interventions, Therapeutics, Assessment, and Line of Care. This approach aligns with international best practices and is compatible with existing tools such as SBAR [4,9,10].

The ABC-VITAL model explicitly recognizes that shift handover requires more than clinical precision; it demands a shared understanding of team context, workload management, and alignment with institutional safety goals [6-8]. It is particularly suited for EDs operating under conditions of time constraints, variable patient flow, and high clinical responsibility.

## Technical report

Shift handovers in ED are critical transitional moments that directly impact patient safety, clinical continuity, and team performance. Unlike ward-based settings, EDs operate in environments of constant flow, cognitive overload, and rapidly evolving patient conditions. In such high-pressure scenarios, any omission, miscommunication, or ambiguity during shift transitions can lead to diagnostic delays, duplicated interventions, or adverse outcomes [1-3]. The handover process in the ED must therefore integrate both the transmission of clinical data and the transfer of accountability, leadership awareness, and anticipatory guidance [4,5].

To address this challenge, we designed a structured and context-sensitive model called the ABC-VITAL for ED handover, composed of three operational domains: Assistance (A), Balance (B), and Closure (C). This model was developed through a narrative literature review of international references focused on communication, safety, human factors, and team dynamics in emergency medicine. A multidisciplinary group of experts in emergency care, health management, and medical education iteratively refined the model via consensus, incorporating both empirical evidence and experiential knowledge from high-complexity emergency units [2,6-8].

Assistance (A)

**Figure 1:**
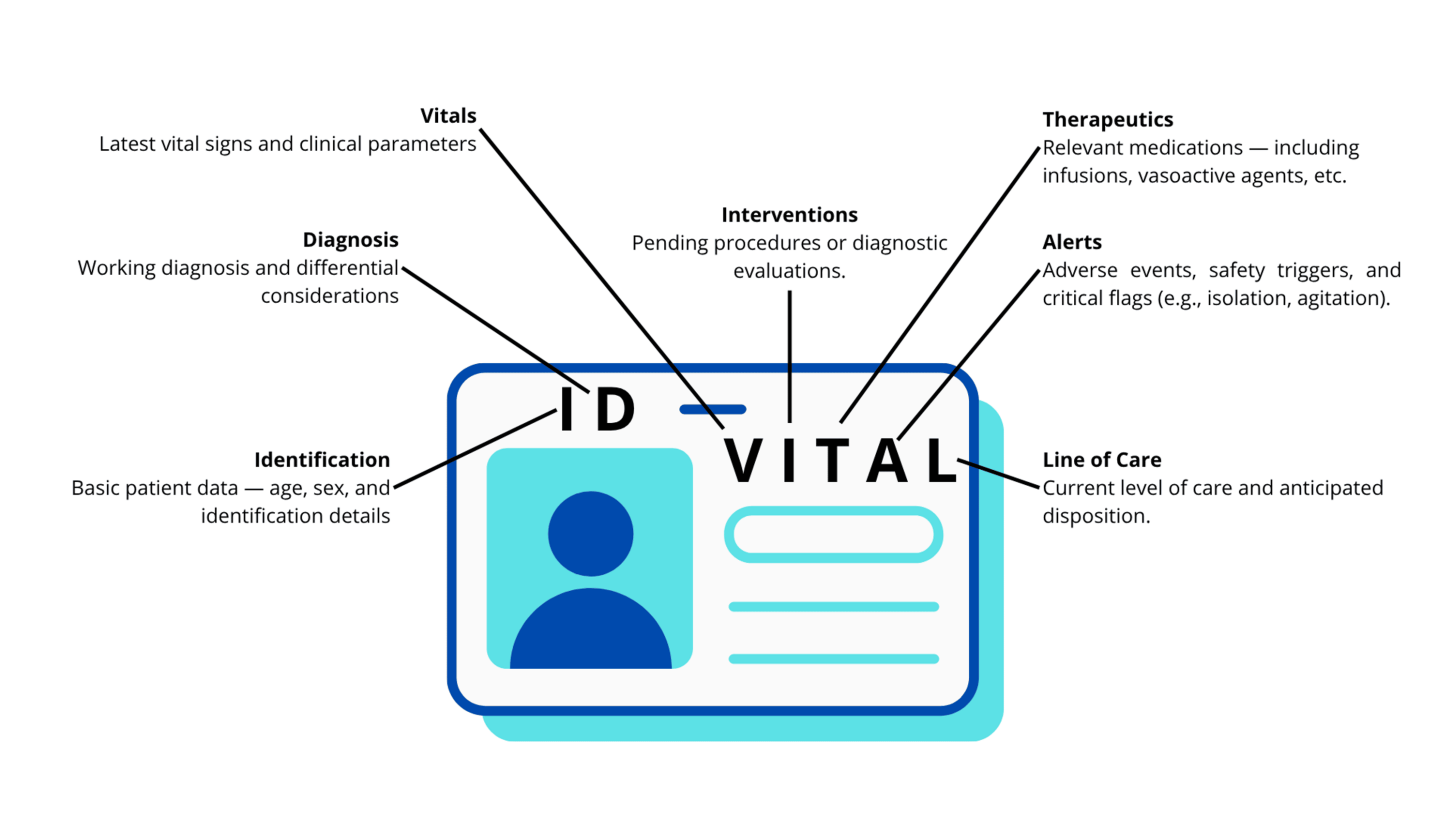
ID-VITAL mnemonic for structured clinical handovers This visual representation summarizes the seven core elements of the ID-VITAL mnemonic, a concise tool for organizing key patient information during ED shift changes. It includes Identification, Diagnosis, Vital Signs, Interventions, Therapeutics, Alerts, and Line of Care. Each component captures essential clinical data to ensure safe and coherent continuity of care.

This domain focuses on the clinical content of the shift handover. It introduces the ID-VITAL mnemonic, a structured tool that captures the essential elements for patient-centered continuity of care. Its components are synthesized in a compact visual aid (Figure 1), designed to support rapid comprehension and ensure the transmission of high-priority clinical information during transitions of care. ID-VITAL serves as an anchor checklist that ensures high-quality, standardized, and comprehensive clinical handovers, reducing omission errors and enabling rapid situational awareness for incoming staff. A customizable template with adaptable examples is available in the Appendix to facilitate implementation and local integration.

Balance (B)

This domain addresses the human and emotional dimensions of shift transition. Emergency physicians frequently accumulate high cognitive and emotional burdens due to unpredictable workloads, ethical dilemmas, and incomplete clinical trajectories. In this context, “balance” involves promoting psychological safety through open dialogue and mutual support, recognizing early signs of team overload, such as unresolved cases, overcrowding, or pending admissions, and encouraging a moment of emotional closure at the end of each shift, particularly when facing complex decisions or adverse outcomes. It also reinforces a shared commitment to collegial accountability and mutual respect during transitions. This component is grounded in literature on leadership under pressure, situational awareness, and the role of emotion in team performance in emergency medicine [6-8].

Closure (C)

The final domain emphasizes operational and cognitive synchronization across the team. Beyond confirming pending tasks, clinical plans, and the transfer of accountability, effective closure requires contextual awareness, both of the macro-environment (external events and institutional dynamics) and the micro-environment within the ED. This phase encourages anticipatory thinking and adaptive reasoning, allowing teams to integrate clinical information with evolving operational realities. Structured transitions, standardized yet flexible, become strategic opportunities to promote leadership, reinforce shared responsibility, and foster team cohesion. Ultimately, consistent handover practices can reduce the need for reactive correction meetings, reinforcing the principle that daily clarity builds long-term resilience [3,6,9,10]. This integrative process fosters adaptive leadership and reinforces continuity in evolving clinical landscapes. The practical implementation of the ABC-VITAL model is intentionally adaptable. Each ED may define the depth and duration of the handover according to its operational tempo. In many settings, team leaders establish the daily work plan immediately after the closure phase, using it as an opportunity to coordinate priorities and clarify the distribution of tasks for the day. This flexible approach ensures that the model strengthens communication and alignment without extending the duration of the handover.

## Discussion

Despite the widespread promotion of structured handover tools in recent decades, the implementation gap remains substantial, particularly in EDs, where time constraints, patient turnover, and variable team compositions challenge the feasibility of rigid protocols. The ABC-VITAL model was developed in direct response to these operational realities, offering a flexible, low-complexity, and context-sensitive approach that centers both team dynamics and patient safety. Unlike existing tools that primarily focus on communication structure or clinical summaries, ABC-VITAL incorporates emotional, ethical, and operational components often neglected in routine handovers.

While this technical report describes the conceptual foundations of the ABC-VITAL model, its implementation and measurable impact remain the focus of future studies. Assessing its influence on communication efficiency, team performance, and safety outcomes will provide the necessary evidence to support its wider adoption. Several international efforts have sought to standardize shift handovers in emergency care, including the ABC framework by Farhan et al. [2], the ED-VITAL mnemonic by Kwok et al. [3], the dINAMO checklist by Rüdiger-Stürchler et al. [11], and SBAR-based communication models [4,12,13]. While these approaches have contributed valuable insights, the present model was developed independently and is not a derivative of any single existing tool. Instead, it integrates selected strengths from prior literature into a novel and context-sensitive framework. What distinguishes this model is its multidimensional structure, encompassing clinical, operational, ethical, and emotional components, which are often overlooked or underdeveloped in prior tools. Its strength lies not only in the content transmitted but also in the way it frames the shift handover as a pedagogical, leadership, and safety-critical act embedded within real-time emergency care dynamics.

**Figure 2:**
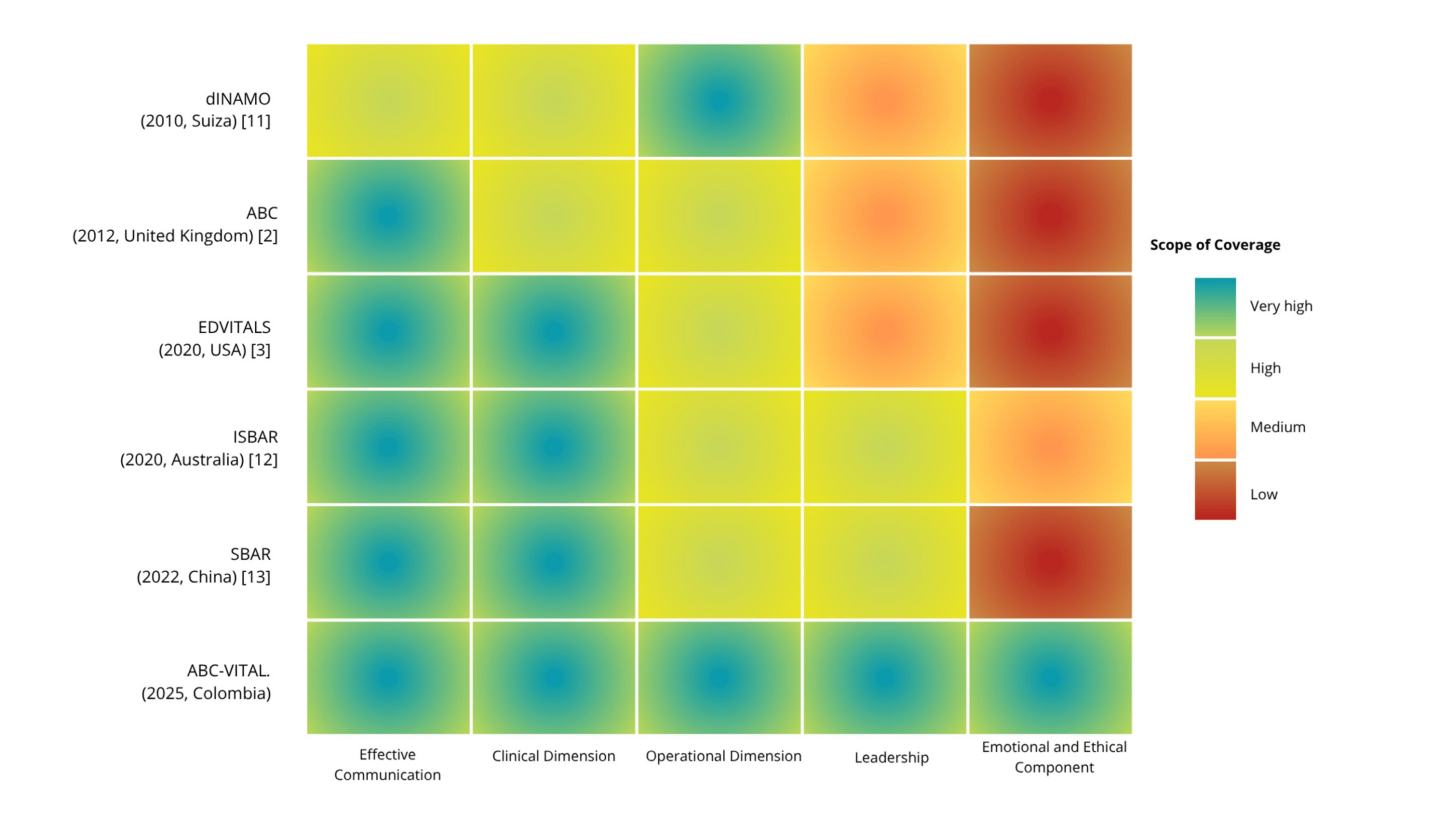
Comparative coverage of handover frameworks across core domains This heat map displays the scope of coverage for six clinical handover frameworks, such as dINAMO [11], ABC [2], EDVITALS [3], ISBAR [12], SBAR [13], and ABC-VITAL, across five essential domains: Effective Communication, Clinical Dimension, Operational Dimension, Leadership, and Emotional and Ethical Component. Color intensity reflects coverage levels, ranging from low (red) to very high (blue-green). The ABC-VITAL model demonstrates the most balanced and comprehensive integration across all domains.

Figure 2 presents a comparative heat map of widely adopted handover strategies. While tools such as SBAR, ISBAR, and EDVITALS demonstrate high coverage in effective communication and basic clinical data, they tend to underrepresent dimensions such as leadership, operational clarity, and emotional integration. ABC-VITAL stands out for its broader scope, particularly in its deliberate inclusion of ethical considerations, team cohesion, and decision-relevant prioritization. This integrative perspective reflects the complex, high-pressure environment of EDs and responds to the multidimensional needs of handover practice.

**Figure 3:**
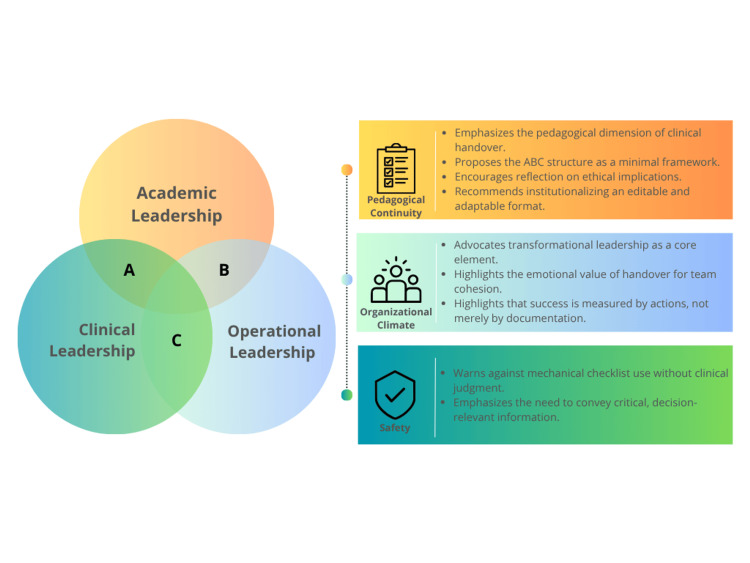
Expert knowledge box: intersecting domains of handover leadership This Venn diagram illustrates the convergence of three core leadership dimensions essential to high-quality shift handovers: academic leadership (continuity and structured reasoning), clinical leadership (patient safety and prioritization), and operational leadership (organizational climate and team performance). Their integration represents the foundation for a comprehensive, adaptive, and context-sensitive handover process.

The development of the ABC-VITAL model was not a purely conceptual exercise but rather the result of a multidisciplinary reflection grounded in frontline experience. Its structure emerged from the convergence of distinct but complementary domains of expertise shared by the authors, including emergency care delivery, clinical leadership, healthcare management, and patient safety. This integration of knowledge is not incidental; it reflects the complex reality of shift handovers, where clinical reasoning, organizational awareness, and human dynamics coexist in time-sensitive contexts. This convergence is visually synthesized in the expert knowledge box (Figure 3), which illustrates the interdependent domains shaping effective handover leadership.

Ultimately, the strength of ABC-VITAL lies not only in its technical structure but also in its capacity to promote cultural transformation. When applied consistently, the model can reduce reliance on retrospective error reviews and exhausting feedback sessions by embedding clarity, accountability, and teamwork into the daily routine. As such, it transforms handovers from a logistical requirement into a deliberate act of leadership and foresight.

Future directions

The ABC-VITAL model offers a practical framework for enhancing shift handovers in ED. Future steps include piloting the model across diverse ED settings, developing standardized training modules for implementation, and evaluating its impact on team performance and patient safety outcomes. Additionally, multicenter validation and contextual adaptation across low- and middle-income countries may expand its applicability and contribute to global efforts in emergency care quality improvement.

## Conclusions

The ABC-VITAL model for shift handovers in ED offers a structured, scalable, and clinically grounded approach that integrates core domains of assistance, team balance, and closure. By embedding the ID-VITAL mnemonic within the clinical component and emphasizing human factors and contextual awareness across transitions, the model transcends traditional checklist paradigms. It fosters operational safety, cognitive clarity, and emotional accountability, which are key pillars for sustaining high-performance teams in dynamic and resource-limited settings. Its flexibility allows for local adaptation without compromising core principles, making it a viable tool for institutional implementation and future research.

## Appendices

**Table 1:** Clinical handover format with examples: ID-VITAL This tool is designed to support structured shift handovers in emergency departments. The mnemonic ID-VITAL stands for: Identification, Diagnosis, Vital signs, Interventions, Therapies, Alerts, and Line of care. It incorporates clinical, operational, and safety-relevant information. The table presents six illustrative cases from general and specialized emergency services. This tool does not replace institutional documentation systems but serves as a flexible aid for verbal or written clinical communication during shift transitions. ASPECTS, Alberta Stroke Program Early CT Score; COPD, chronic obstructive pulmonary disease; ECOG, Eastern Cooperative Oncology Group; IMCU, intermediate care unit; mRS, Modified Rankin Scale; NIMV, non-invasive mechanical ventilation; NIHSS, National Institutes of Health Stroke Scale; PO, by mouth; PRN, as needed; RA, room air; s/p, status post; tPA, tissue plasminogen activator; TBI, traumatic brain injury; VAS, Visual Analogue Scale

Identification	Diagnosis	Vital Signs	Interventions	Therapies	Alerts	Line of Care
Female, 64 y/o. Currently in Observation Unit M01	Urosepsis with initial hypotension and fever	Now afebrile, BP 110/65, HR 88, RR 18, SpO2 96% RA	Blood cultures drawn, Foley catheter placed, urine sample pending	Ceftriaxone IV, maintenance fluids	Frailty, living alone, requires social work follow-up	Interfacility transfer arranged; pending ambulance availability
Male, 72 y/o. Admitted post-op, bed H09	Postoperative delirium, s/p hip arthroplasty	Fluctuating consciousness, BP 135/80, HR 95, afebrile	Head CT performed-small parietal ICH; fall occurred during prior shift	IV morphine PRN, IV fluids, and delirium precautions in place	High fall risk; family unaware of incident; requires escalation	Transfer to IMCU requested; neurosurgery notified
Male, 58 y/o. Resuscitation Bay 01	Acute-on-chronic COPD with hypercapnic respiratory failure	On NIMV: RR 22, SpO2 92%, pH 7.33, PaCO2 58 mmHg	Arterial blood gas obtained, sputum sample pending	NIMV, IV methylprednisolone, nebulized ipratropium/albuterol	Poor adherence to meds; companion left during stay	Awaiting ICU bed; already accepted by the intensivist
Female, 61 y/o. Isolation Room 01	Advanced gastric carcinoma with new respiratory symptoms	RR 28, SpO2 88% on nasal cannula, somnolent but arousable	Palliative care consulted; chest X-ray pending	Continuous IV infusion of morphine and midazolam	No advance directives; family requesting deep sedation	Awaiting ethics team input for palliative sedation protocol
Female, 75 y/o. Critical Area 02	Acute ischemic stroke, NIHSS 14, ASPECTS 8	BP 150/90, HR 78, NIHSS unchanged post-tPA	IV thrombolysis performed two hours ago; angio pending	IV fluids, neuro checks q15min, bedrest	Minor groin site bleeding; controlled with compression	Transfer to the angiography unit pending bed turnover
Male, 55 y/o. Intermediate Zone 01	Suspected NSTEMI with atypical presentation	BP 120/80, HR 72, pain-free, normal ECG, first trop negative	Serial ECG monitoring, second troponin ordered	Aspirin 300 mg PO, atorvastatin 80 mg PO	Highly anxious about discharge; low social support	Awaiting the second troponin to determine admission vs. discharge
